# Differenzialdiagnose chronischer Abdominalschmerzen – Zufallsbefund einer auffälligen Leberläsion bei einem 15-jährigen Mädchen mit Bauchschmerzen

**DOI:** 10.1007/s00104-023-02009-2

**Published:** 2024-01-02

**Authors:** Tristan Wagner, Wasim Kamel, Raphael Stier, Thorsten Persigehl, Rabi Datta, Christiane Bruns, Dirk Stippel, Michael Thomas

**Affiliations:** 1 Klinik für Allgemein‑, Viszeral‑, Tumor- und Transplantationschirurgie, Universitätsklinikum Köln, Köln, Deutschland; 2 Institut für Diagnostische und Interventionelle Radiologie, Universitätsklinikum Köln, Köln, Deutschland

**Keywords:** Lebertumor, Inflammatorischer Pseudotumor, Appendizitis, Bauchschmerzen

## Anamnese

Ein 15-jähriges Mädchen stellte sich bei persistierenden Bauchschmerzen und Übelkeit in Begleitung ihrer Mutter in unserer Ambulanz vor. Die Beschwerden bestünden bereits seit vielen Jahren. Die Bauchschmerzen seien auch nachts sowie vor dem Stuhlgang vorhanden. Der Stuhlgang sei normal und regelmäßig. Gewichtsverlust und klassische B‑Symptomatik werden verneint. In der Vorgeschichte seien im Rahmen mehrere klinischen Vorstellungen der Verdacht auf eine Fruktoseintoleranz gestellt worden. Beim Verdacht auf eine lymphozytäre Kolitis in der Vorgeschichte erfolgte, nach diagnostischer Sicherung mittels Koloskopie, eine Steroidtherapie. Zu diesem Zeitpunkt bestanden keine akuten abdominellen Beschwerden. Die Patientin ist psychisch und physisch altersgerecht normal entwickelt. Die familiäre Anamnese war unauffällig.

## Klinischer Befund

Die körperliche Untersuchung ergab keinen wegweisenden Befund. Körperhöhe: 163,8 cm, Gewicht: 50,9 kg, BMI: 19,0 kg/m^2^, Körperoberfläche: 1,5 m^2^. Der Bauch imponierte klinisch weich, jedoch diffus druckschmerzhaft, betont in den unteren Quadranten, jedoch ohne Abwehrspannung. Die Patientin befand sich in einem guten Ernährungszustand, zeigte keinen Ikterus oder pathologische Hautveränderungen. Die klassischen Appendizitiszeichen und das Murphy-Zeichen waren negativ.

Zur weiteren Abklärung erfolgte zunächst eine sonographische Untersuchung. Dabei zeigte sich kein Anhalt für eine akute Appendizitis oder gynäkologische Auffälligkeiten. Jedoch war eine auffällige echoarme Struktur im Lebersegment I (lma 300–310) sowie ein akzentuierter Lymphknoten (0,8 cm in der Kurzachse) zu sehen, woraufhin bei systemisch-laborchemisch deutlich erhöhten Inflammationszeichen eine MRT-Untersuchung indiziert wurde.

## Laborkonstellation

CRP 120,8 mg/l; Procalcitonin < 0,02 µg/l; Leukozyten 8,48 10*9/l; Hämoglobin 11,0 g/dl; Hämatokrit 34 %; Erythrozyten 3,92 × 10^6^/µl, Bilirubin < 0,01 mmol/l; ASAT 32 U/l; ALAT 44 U/l; AP 121 U/l; GGT 63 U/l.

## Mikrobiologie

- 2‑mal periphere Blutkulturen: kein Erregernachweis
- Quantiferontest: negativ
- Lues-Serologie: negativ
- Toxoplasmose-Serologie: negativ
- Brucellose-Serologie: negativ
- *Bartonella*-Serologie: negativ
- β‑D-Glykan: negativ

## Bildgebende Untersuchung (MRT; Abb. 1)

Hier zeigte sich initial eine hyperperfundierte Raumforderung (ca. 2,5 × 2 0,0 × 3,4 cm) im Lebersegment I mit zentralem Wash-out. Eine etwas kleinere ebenfalls hyperperfundierte Läsion mit zentralem Wash-out war auch in Segment VI zu sehen. Es bestand bildmorphologisch kein Anhalt auf eine Kolitis, nebenbefundlich zeigten sich am ehesten zyklusbedingt zystische Ovarien beidseits.

**Figure 1:**
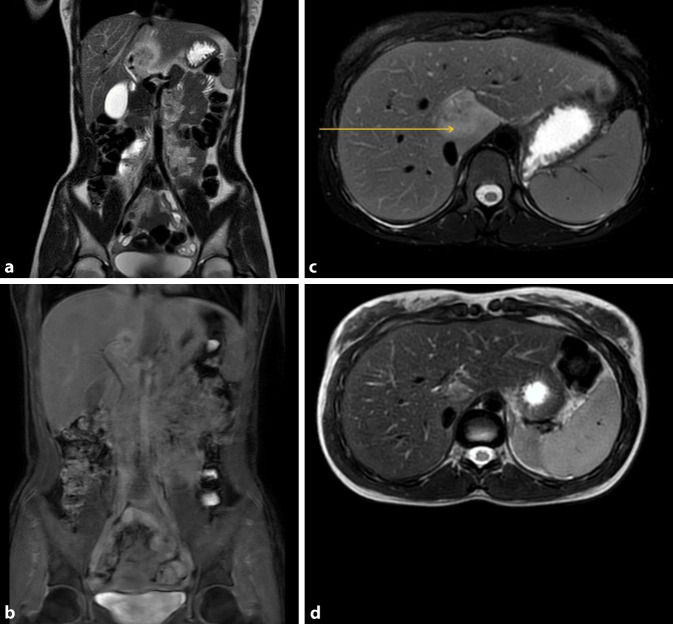

## Weiteres Prozedere

Es erfolgte die Virusdiagnostik (Hepatitis A, B, C, D) mit negativen Befunden sowie eine ebenfalls unauffällige Stuhluntersuchung. Parasitäre Erkrankungen konnten serologisch ausgeschlossen werden. Bei weiterhin bestehender unklarer Dignität der Leberraumforderung wurde die stationäre Aufnahme zur weiteren Abklärung geplant. Der Fall wurde in unserer interdisziplinären Lebertumorkonferenz vorgestellt. Hier wurde bei unklarer Dignität der Leberraumforderungen die Empfehlung zur CT-gesteuerten histologischen Sicherung empfohlen.

## Wie lautet Ihre Diagnose?

Histologischer Befund: In der Leberbiopsie in Segment I zeigten sich eine Gewebsnekrose und eitrige sowie granulomatöse Begleitentzündung. Eine infektiöse Genese, die mit Nekrosen und begleitenden Epitheloidzellgranulomen einhergeht, kommt hier in Betracht (z. B. Listeriose, Brucellose, weniger wahrscheinlich vom histomorphologischen Aspekt eine Tuberkulose).

Eine mikrobiologische Abklärung ist notwendig. Favorisiert wird eine infektiöse Genese. Kein Anhalt für Malignität.

Die mikrobiologischen Untersuchungen der Biopsie waren jedoch ohne pathologische Befunde.

Leberbiopsie:

- Listerien-PCR: negativ
- *Mycobacterium*-Spezies-PCR: negativ
- *Chlamydia-trachomatis*-PCR: negativ
- *Bartonella*-PCR: negativ
- *Brucella*-PCR: negativ
- *S. aureus*-PCR: negativ
- Entamöben: negativ
- Echinokokken: negativ

**Diagnose:** entzündlicher Pseudotumor der Leber (IPT)

## Definition

Beim entzündlichen hepatischen Pseudotumor (IHP) handelt es sich um eine seltene, jedoch gutartige Läsion der Leber unklarer Genese, die gehäuft bei Kindern und jungen Erwachsenen vorkommt [1]. Erstmalig beschrieben wurde der IHP 1953 durch Pack und Baker im Rahmen einer histopathologischen Aufarbeitung eines unklaren Tumors des rechten Leberlappens [2]. Aufgrund seiner bildgebenden Eigenschaften und fehlender sicherer Diagnosekriterien kann er mit malignen Tumoren verwechselt werden. Entzündliche hepatische Pseudotumoren treten meist sporadisch auf, ätiologisch werden jedoch unter anderem Infektionen oder Autoimmunerkrankungen diskutiert [3]. Die eindeutige Diagnose eines entzündlichen hepatischen Pseudotumors bedarf einer bioptischen Sicherung und konsekutiv einer histopathologischen Aufarbeitung. Das pathologische Bild des IHP imponiert dabei insgesamt heterogen. Zen et al. teilten den IHP in zwei Typen ein: 1) den fibrohistiozytischen Typen, der häufig im peripheren Leberparenchym vorkommt, und 2) den lymphoplasmatischen Typ, der insbesondere im Bereich des Leberhilus entsteht und möglicherweise zu den IgG 4-assoziierten Erkrankungen gezählt werden kann [4].

## Therapie und Verlauf

Die Therapie des entzündlichen Leberpseudotumors hängt von Faktoren wie der Tumorgröße, den Symptomen und dem gesundheitlichen Zustand des Patienten ab. Zu den Therapiemöglichkeiten gehören die Observation bei asymptomatischen kleinen Läsionen, die medikamentöse Behandlung zur Entzündungshemmung und Schmerzlinderung, interventionelle Verfahren wie die Drainage oder Embolisation bei symptomatischen Fällen und in seltenen Fällen die chirurgische Entfernung. Eine regelmäßige Nachsorge ist entscheidend, um ein Wiederauftreten oder etwaige Komplikationen zu identifizieren [4].

Die vorgestellte Patientin wurde zunächst empirisch antibiotisch mit Piperazillin/Tazobaktam anbehandelt. Bei stetig fallenden Infektparametern konnte die Antibiose zeitgerecht entfernt werden. Hiernach zeigten sich eine Normalisierung der Infektwerte und ein regredienter Residualbefund in der MRT. Die Patientin berichtete hierzu passend über eine Besserung der abdominellen Schmerzen. Der Residualbefund soll in regelmäßigen Verlaufsuntersuchungen mittels Schnittbildgebung kontrolliert werden. Bei der jungen Patientin wurde als Modalität die MRT gewählt und aktuell ein 3‑monatiges Intervall festgelegt.

## Fazit für die Praxis

- In der modernen Chirurgie ist die abdominale MRT-Untersuchung und interdisziplinäre Tumorkonferenzen von entscheidender Bedeutung, da sie die diagnostische Genauigkeit verbessern, eine umfassende Behandlungsplanung ermöglichen, minimal-invasive Techniken unterstützen, das chirurgische Risiko abschätzen, eine personalisierte Behandlung ermöglichen und zu verbesserten Patientenergebnissen bei der Behandlung abdominaler Tumoren und verwandter Erkrankungen beitragen.
- Die rechtzeitige Erkennung und adäquate Behandlung raumfordernder Läsionen in der Leber ist von entscheidender Bedeutung. Diffuse Bauchschmerzen sollten daher sorgfältig abgeklärt werden, und es sollte eine gründliche Untersuchung, einschließlich Bildgebung und gegebenenfalls eine Biopsie, durchgeführt werden, um die genaue Ursache zu ermitteln und eine angemessene Behandlung zu gewährleisten.
